# Characterization of oxidative status in maize protoplasts under temperature and saline-alkali stresses

**DOI:** 10.1186/s12870-026-09311-1

**Published:** 2026-06-24

**Authors:** Wenzhu Wang, Juan Li, Xia Hu, Zhigang Liu, Darun Cai, Xiaorong Li, Tengkun Nie, Bo Li, Xunji Chen, Yunlong Zhai, Guo Chen

**Affiliations:** 1 College of Agriculture, Tarim University, Alar, 843300 China; 2 Xinjiang Key Laboratory of Crop Biotechnology, Biological Breeding Laboratory, Xinjiang Academy of Agricultural Sciences, Urumqi, 830091 China

**Keywords:** Maize, Protoplast, High temperature, Low temperature, Saline–alkali, Functional verification

## Abstract

**Background:**

Protoplasts have emerged as a powerful model system in plant functional genomics, offering significant utility in functional gene analysis, protein interaction studies, and transient expression platforms for gene editing. Despite their versatility, inherent limitations restrict their broader application, highlighting the need for systematic investigations into their responses to abiotic stressors, such as temperature fluctuations and saline-alkali conditions (200 mM saline mixture: 170mM NaCl and 30mM Na_2_CO_3_, pH = 9.1).

**Results:**

In this study, we comprehensively examined the effects of varying temperatures and saline-alkali stress on the integrity, viability, and reactive oxygen species (ROS) metabolism of maize protoplasts. Key markers of oxidative stress-including ROS accumulation, lipid peroxidation (measured as malondialdehyde, MDA), antioxidant enzyme activity (superoxide dismutase, SOD), and hydrogen peroxide (H_2_O_2_) levels-were quantified to assess the oxidative stress response. Protoplasts maintained at 4 °C demonstrated enhanced stability and antioxidant capacity, preserving cell viability and endogenous protein integrity for up to 16 h. Conversely, exposure to 37 °C significantly compromised protoplast viability, while incubation at 28 °C exerted minimal effects within 16 h.

**Conclusions:**

Our study investigated the effects of various temperature stresses and salt-alkali stress on maize protoplasts. The results demonstrated that both temperature and salt-alkali stress significantly impacted protoplast production, viability, and the expression of endogenous proteins. These findings not only characterize the redox response of maize protoplasts, but also provide guidance for protoplast isolation and other procedures: 4 °C is suitable for short-term maintenance, 25–28 °C for routine functional assays, and 37 °C should be avoided. These findings provide valuable insights into the stress responses of protoplasts and establish a foundation for future research aimed at improving plant stress tolerance through protoplast-based techniques.

**Supplementary Information:**

The online version contains supplementary material available at https://doi.org/10.1186/s12870-026-09311-1.

## Background

Protoplasts, plant cells stripped of their cell walls and enclosed solely by the plasma membrane, consist of cytoplasm, a nucleus, and various organelles. These cellular systems have become indispensable in plant biotechnology, facilitating applications such as genetic transformation [1], somatic hybridization, germplasm conservation, mutant selection, plant cell reprogramming and transient gene expression [2]. Due to the absence of a cell wall, protoplasts are more susceptible to the uptake of external genetic material, such as DNA, making them ideal for recipients for plant genetic transformation [3]. Furthermore, protoplasts have potential in the fields of germplasm storage and biotechnological research, among others. However, temperature is a critical environmental factor that influences the vitality and regenerative capacity of protoplasts, which is exhibited by their high sensitivity to fluctuations in temperature. Furthermore, suboptimal temperature conditions can cause impaired enzymatic activity, cell membrane damage, and metabolic imbalances, all of which impact the s the downstream use of protoplasts [4]. Previous research indicated that the optimal temperature range for protoplasts typically falls between 25 ℃ and 30 ℃ [5].

In addition, protoplasts are highly sensitive to environmental perturbations, particularly temperature fluctuations. Suboptimal thermal conditions can disrupt enzymatic activity, impair membrane integrity, and alter metabolic processes, ultimately compromising protoplast viability and their utility in downstream applications [6]. Previous transient expression assays were conducted exclusively at 25 °C [7]. Therefore, the present study uses 25 °C as the reference temperature and extends the incubation range to 4–37 °C to quantify temperature-specific oxidative responses. Temperatures that deviate ≥ 10 °C from the species-specific in vitro optimum (usually 24–28 °C for temperate crops) are generally considered stressful for isolated protoplasts. At ≤ 4 °C, membrane rigidification and microtubule depolymerisation activate cold-responsive MAPKs within minutes, leading to Ca^2+^-dependent ROS bursts that compromise viability [8]. Conversely, ≥ 35 °C causes membrane fluidisation, protein denaturation and cytoskeletal disintegration; maize protoplasts lose > 50% viability within 2 h at 37 °C, accompanied by a 3-fold increase in H_2_O_2_ and lipid peroxidation [9]. The present study therefore adopted 4 °C and 37 °C as clearly sub- and supra-optimal extremes to dissect temperature-specific oxidative responses, while 28 °C served as the non-stress control.

Beyond their biotechnological applications, protoplasts are valuable tools for investigating fundamental biological responses to abiotic stress, including protein signalling, ion fluxes, reactive oxygen species (ROS) production, and plasma membrane dynamics [10]. Superoxide dismutase (SOD), a cornerstone of the plant antioxidant defense system, serves as the first line of protection against ROS generated under abiotic stresses [11]. Extensive studies have demonstrated SOD’s critical role in mitigating physiological and biochemical damage caused by adversities such as drought [12], salinity [13], and cold temperatures [14]. Overexpression of SOD genes can enhance stress tolerance by regulating ROS homeostasis, thereby preserving cellular stability and resilience to environmental challenges like drought, salinity, and extreme temperatures [15]. Hydrogen peroxide (H_2_O_2_), a ubiquitous and highly diffusible reactive oxygen species (ROS), plays a dual role in plant cells. At low concentrations, it functions as a signaling molecule that regulates processes such as proliferation, differentiation, energy metabolism, and gene expression [16]. Additionally, its capacity to diffuse across cellular compartments amplifies its impact under stress conditions [17]. Understanding H_2_O_2_-mediated stress tolerance mechanisms is vital for developing biotechnological strategies to improve crop resilience [16].

Saline-alkali stress, a major abiotic constraint on global agriculture, severely impacts plant cell integrity and functionality. Investigating protoplast responses to saline-alkali conditions offers critical insights into the mechanisms underpinning tolerance to such stress [18]. The unique structure of protoplasts enables detailed studies of plasma membrane transporter regulation under saline-alkali stress [19], while salt and alkali exposure triggers gene expression changes linked to ion transport, antioxidant defense, osmotic adjustment, and signal transduction [20]. These attributes make protoplasts an ideal system for dissecting cellular adaptation strategies. Maize (Zea mays L.), a well-established model organism, has garnered significant attention in functional genomics, translational genomics, and epigenetics research [21–23].

Temperature-dependent oxidative physiology has been studied almost exclusively in dicot protoplasts (Arabidopsis thaliana, Nicotiana benthamiana) [24], and a quantitative response framework for monocot protoplasts remains limited. In maize, the first high-yield mesophyll protoplast isolation protocol was established only three decades ago [25]. Subsequent refinements have focused on maximizing transfection efficiency (> 90% at 25–28 °C, 6–12 h) [26, 27]; however, viability declines sharply below 24 °C or above 32 °C, and long-term maintenance is limited by temperature-induced reactive oxygen species (ROS) bursts that trigger premature senescence [28]. A systematic, parallel comparative analysis of low- versus high-temperature effects on ROS metabolism, lipid peroxidation, and antioxidant enzyme activity in maize protoplasts is therefore still lacking. In this study, we explored the adaptive responses of maize protoplasts to high temperature, low temperature, and saline-alkali stress. Protoplast isolation provides a robust platform for transient gene function analysis, while our findings offer practical guidance for refining protoplast preparation protocols, optimizing culture conditions, and enhancing the efficiency of protoplast-based applications in plant biotechnology. Specifically, this study transcends the simplistic notion that low temperature preserves protoplasts and establishes a practical temperature - time window for the manipulation of maize protoplasts.

## Results

### Effect of various incubation temperatures on protoplasts

**Fig. 1:**
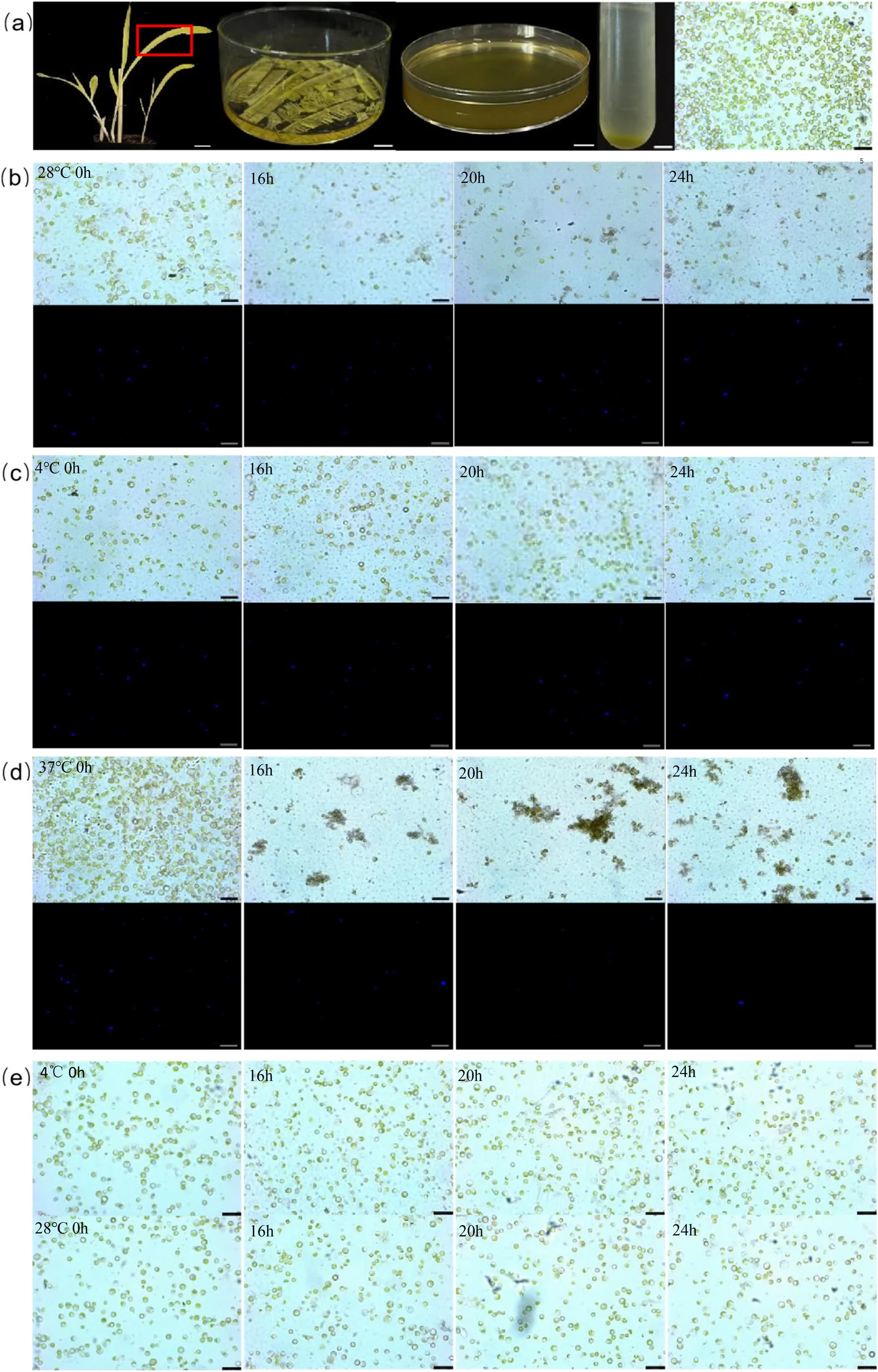
Time-Course Visualization of Temperature- and Saline–Alkali-Induced Stress Responses in Maize Protoplasts. **a** Overview of the experimental procedures for isolating maize protoplasts. The scale of the extraction flowchart is 2 cm, while the scale of the cell chart is 50 µm. **b** Protoplast production was observed after incubation at 28 °C for 0 h, 16 h, 20 h, and 24 h. Scale bars = 50 µm. **c** Protoplast production was observed after incubation at 4 °C for 0 h, 16 h, 20 h, and 24 h. Scale bars = 50 µm. **d** Protoplast production was observed after incubation at 37 °C for 0 h, 16 h, 20 h, and 24 h. Scale bars = 50 µm. **e** Microscopic images of protoplasts in a mixed solution of NaCl and Na2CO3 (200 mM) were incubated at 4 °C and 28 °C for durations of 0 h, 16 h, 20 h, and 24 h. Scale bars = 50 µm. Temperature labels are indicated on the panels for clarity. Blue dots indicate fragmented or lysed protoplasts. Representative micrographs are shown; the corresponding quantitative data are provided in Fig. 2

**Fig. 2:**
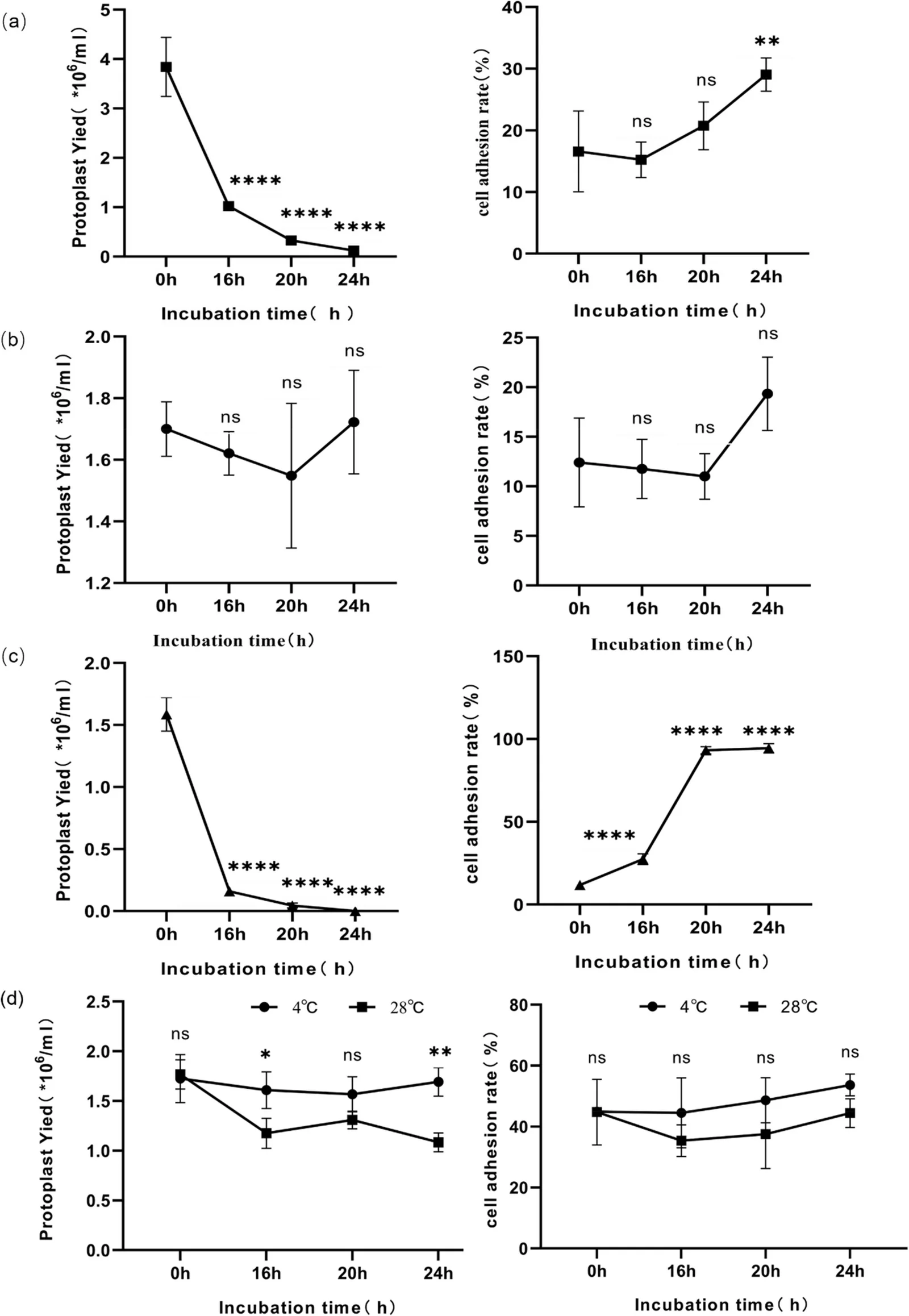
Effect of Various Incubation Temperatures on Protoplasts. **a** Graphs showing cell counts and cell adhesion rates following incubation at 28 °C for 0 h, 16 h, 20 h, and 24 h. **b** Plots of cell counts and cell adhesion rates after incubation at 4 °C for 0 h, 16 h, 20 h, and 24 h. **c** Plots of cell counts and cell adhesion rates after incubation at 37 °C for 0 h, 16 h, 20 h, and 24 h. **d** Plots of protoplast cell numbers and adhesion rates after incubation at 4 °C and 28 °C for 0 h, 16 h, 20 h, and 24 h under stress from a mixed saline and alkaline solution. Data significance is indicated as follows: ns (*P* > 0.05), * (*P* < 0.05), ** (*P* < 0.01), *** (*P* < 0.001), **** (*P* < 0.0001) (GraphPad format). Data are presented as mean ± SD from three independent biological replicates

Throughout this study, “protoplast concentration” refers to the number of viable, cell-wall-free protoplasts per mL determined after enzymatic isolation, and t = 0 h denotes freshly isolated protoplasts before temperature incubation. Immediately after isolation (t = 0 h, no incubation) the initial protoplast concentration was 3.84 × 10^6^ mL^− 1^ with an inter-cellular adhesion rate (percentage of protoplasts in ≥ 3-cell clumps) of 12.39%. Subsequent incubation (in WI medium at the indicated temperature) for 16 h at 28 °C caused limited changes relative to the t = 0 h control. After 16 h of incubation, the protoplast concentration decreased to 1.02 × 10^6^ mL^− 1^, and the intercellular adhesion rate slightly reduced to 12.20%. As the incubation time was further extended, both protoplast concentration and activity continued to decline, while the intercellular adhesion rate increased. Specifically, after 24 h of incubation, the protoplast concentration dropped significantly to 0.12 × 10^6^ mL^− 1^, while the intercellular adhesion rate increased to 31.85% (Figs. 1b and 2a).

These results suggest that, at the optimal growth temperature of 28 °C for maize protoplasts, an incubation period of 16 h had minimal impact on cell activity, integrity, and intercellular adhesion. However, incubation beyond 20 h significantly affected protoplast concentration, integrity, and intercellular adhesion, which may pose challenges for practical applications. This conclusion was drawn from a comparative analysis of protoplast growth and activity at 4 °C, 28 °C, and 37 °C.

When incubated at 37 °C for 16 h, protoplast cells exhibited significant damage compared to the 0 h incubation period. This was evidenced by a substantial reduction in cell count from 1.59 × 10^6^ mL^− 1^ to 0.2 × 10^6^ mL^− 1^, cell clumping, severe fragmentation, and an increase in the intercellular adhesion rate from 9.47% to 27.38%. After 24 h of incubation, the adhesion rate further increased to 94.39% (Figs. 1d and 2c). These findings suggest that exposure to 37 °C is highly detrimental to protoplasts, potentially causing irreversible damage to cellular organelles and significantly hindering their subsequent utilization.

During the 24-hour incubation period, the cell count of maize protoplasts maintained at 4 °C fluctuated between 1.55 × 10^6^ mL^− 1^ and 1.72 × 10^6^ mL^− 1^. After 24 h of incubation, the protoplast concentration reached 1.72 × 10^6^ mL^− 1^ at 4 °C (Fig. 2b), compared to only 0.12 × 10^6^ mL^− 1^ at 28 °C (Fig. 2a). Additionally, the intercellular adhesion rate was 19.34% at 4 °C (Fig. 2b).

NaCl and Na_2_CO_3_ were selected as the salt and alkali components for the stress solution due to their common use in simulating salt-alkali stress conditions in agricultural research. The final concentration of 200 mM in the protoplast suspension was chosen based on preliminary experiments, where this concentration demonstrated significant but non-lethal effects on protoplasts, ensuring observable stress responses without complete cell death. Under saline-alkali stress (200 mM NaCl/Na_2_CO_3_, pH 9.1), protoplasts incubated at 4 °C retained spherical morphology and minimal adhesion throughout 24 h, whereas at 28 °C progressive clumping and membrane blebbing were evident (Fig. 1e).This concentration enables us to study the protoplasts’ stress tolerance and response mechanisms effectively.

The protoplast suspension did not have a specific concentration measurement. Instead, the protoplast concentration was expressed in terms of the number of protoplasts and the intercellular adhesion rate. During incubation, both the protoplast concentration and intercellular adhesion rate exhibited dynamic changes. For instance, prior to incubation at 28 °C, the initial protoplast concentration was 3.84 × 10^6^ cells mL^− 1^, with an intercellular adhesion rate of 12.39%. After 16 h of incubation, the protoplast concentration decreased to 1.02 × 10^6^ cells/mL, and the intercellular adhesion rate slightly declined to 12.20%. As the incubation time was further extended, both protoplast concentration and activity continued to decrease, while the intercellular adhesion rate increased. Specifically, after 24 h of incubation, the protoplast concentration dropped significantly to 0.12 × 10^6^ cells mL^− 1^, while the intercellular adhesion rate rose to 31.85% (Fig. 2a). At 37 °C, protoplast yield declined precipitously from 1.5 × 10^6^ mL^− 1^ at 0 h to near-zero by 24 h, while cell adhesion rate rose sharply from ~ 10% to ~ 95% over the same period (Fig. 2c).

### Effects of different incubation temperatures on oxidative stress indicators in protoplasts

To evaluate the oxidative status and antioxidant capacity of protoplasts at various incubation temperatures, we measured key oxidative stress indicators (ROS, MDA, SOD, and H_2_O_2)_ after incubation periods of 0, 16, 20, and 24 h.

**Fig. 3:**
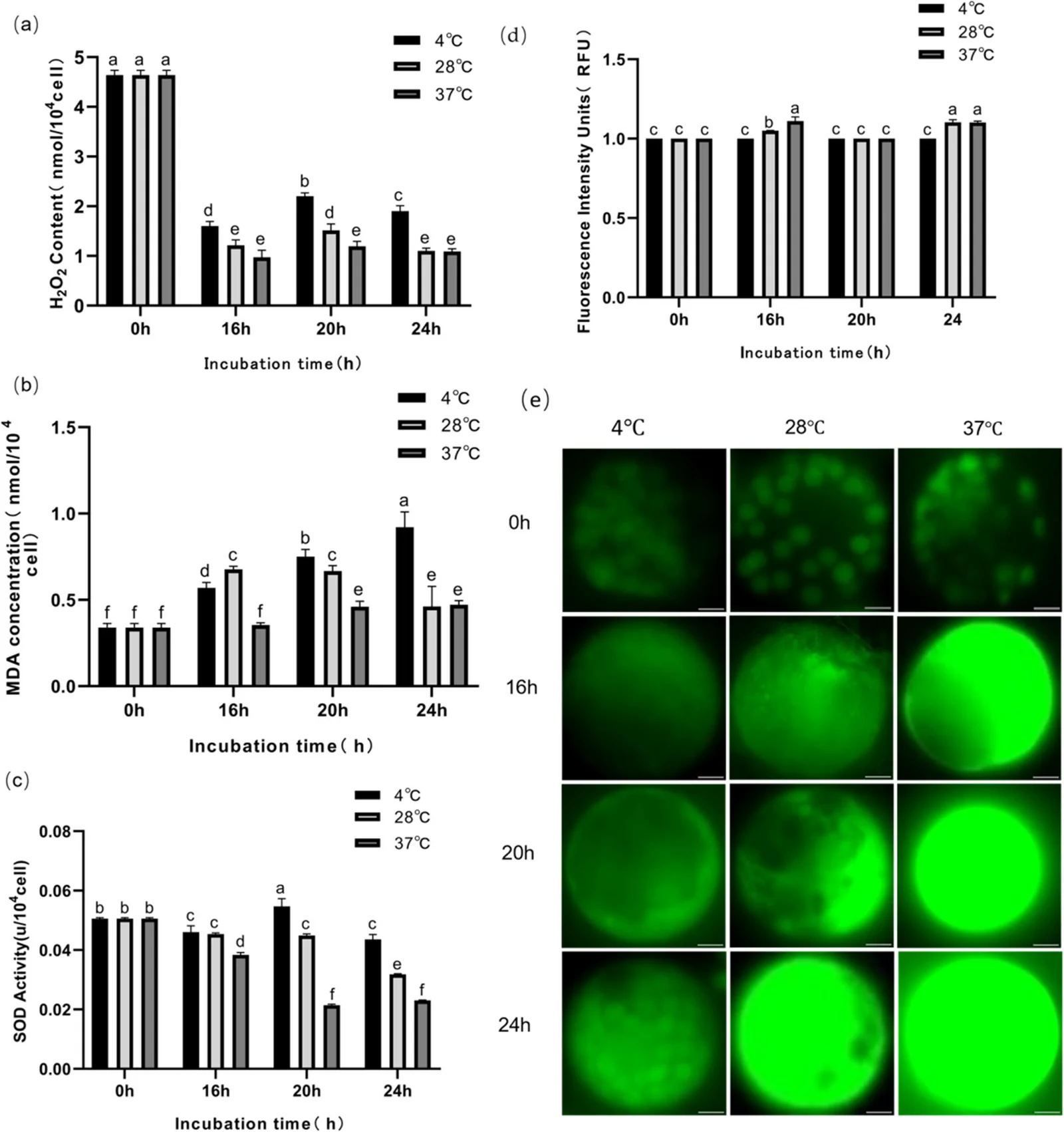
Oxidative Stress Indicators in Maize Protoplasts at Varying Incubation Temperatures The plots depict oxidative stress indicators in maize protoplasts incubated at different temperatures. **a** Levels of hydrogen peroxide (H2O2) in protoplasts under varying temperature stress conditions. **b** Content of malondialdehyde (MDA) in protoplasts under different temperature stress conditions. **c** Activity of superoxide dismutase (SOD) in protoplasts under varying temperature stress conditions. **d** Levels of reactive oxygen species (ROS) using the DCFH-DA probe, measured via spectrophotometry in protoplasts under different temperature stress conditions. **e** Fluorescence intensity of ROS in maize protoplasts after 0, 16, 20, and 24 h of incubation under varying temperature stress conditions, expressed in fluorescence intensity units. Fluorescence microscopy images are provided with a scale bar of 3 μm. Different lowercase letters (**a**, **b**) denote significant differences between groups (*p* < 0.05, two-way ANOVA with Tukey’s post-hoc test). *n* = 3 biological replicates

The levels of MDA, SOD, and H_2_O_2_ were determined using enzyme-linked assays following protoplast lysis after incubation at 4 °C, 28 °C, and 37 °C. At 4 °C, the activity of SOD in protoplasts significantly decreased over time, while H_2_O_2_ levels markedly increased (Fig. 3a). Additionally, MDA content showed a gradual upward trend. Relative to 0 h, SOD decreased to 67.4% at 24 h, whereas H_2_O_2_ and MDA increased by approximately 1.42-fold and 1.28-fold, respectively. These changes may be attributed to the inhibition of overall cellular metabolic activities under low-temperature conditions, leading to reduced SOD synthesis. In contrast, the accumulation of H_2_O_2_ and MDA indicates an imbalance in the cellular antioxidant system.

At 28 °C and 37 °C, the trends in SOD and H_2_O_2_ levels were similar, with both reaching their lowest levels at 16 h before recovering. However, these changes were not statistically significant. This suggests that the cellular antioxidant system was more effective in maintaining balance at these temperatures. When treated at 37 °C, the activity of SOD decreased most significantly, dropping by approximately 58.4% after 20 h of treatment, indicating that high temperature markedly affected the antioxidant capacity of protoplasts(Fig. 3c). In contrast, a slight decrease in MDA content was observed during prolonged incubation at 28 °C. This trend may be attributed to the activation of the cellular antioxidant defense system, balanced metabolic activities, stable intracellular environment, and enhanced MDA scavenging mechanisms. These findings demonstrate that protoplasts exhibit robust adaptive capacity and self-protective mechanisms under relatively mild temperature conditions.

Additionally, we investigated the changes in intracellular reactive oxygen species (ROS) content in protoplasts subjected to prolonged stress at both low and high temperatures. This analysis was based on the measurement of intracellular ROS using the DCFH-DA dye, with fluorescence intensity serving as an indicator of ROS levels. Throughout the 24-hour incubation period, the fluorescence intensity showed minimal variation, remaining consistently around 1.00 RFU. However, significant differences were observed at different incubation temperatures. As depicted in Fig. 3d, at 4 °C, cells gradually acclimated to the low-temperature environment over time, maintaining stable fluorescence intensity. This stability may indicate the presence of a cyclic or repetitive physiological process that helps maintain ROS homeostasis under low-temperature stress. In contrast, at incubation temperatures of 28 °C and 37 °C, the fluorescence intensity change curves were relatively consistent, with a peak observed at 16 h of incubation (Fig. 3e). At 16 h, ROS fluorescence reached approximately 1.87-fold of the 0 h level at 28 °C and 2.96-fold at 37 °C (Fig. 3d). This suggests that, at these higher temperatures, ROS levels initially increase before stabilizing. These findings collectively indicate that temperature significantly influences the intracellular concentrations of ROS, superoxide dismutase (SOD), malondialdehyde (MDA), and hydrogen peroxide (H_2_O_2_), highlighting the importance of temperature in modulating oxidative stress and antioxidant responses in protoplasts.

### Effect of various incubation temperatures on protoplasts under saline-alkaline stress

To further investigate the potential of protoplasts as a model system for studying gene regulation under saline stress, we examined the effects of different incubation temperatures on protoplasts subjected to saline - alkaline stress. The results indicated that the number of protoplasts incubated at 4 °C for 16 and 24 h was significantly higher than that at 28 °C (Fig. 2d). Moreover, at the same temperature and incubation duration, the adhesion rate of protoplasts subjected to saline stress was significantly greater than that of cells without saline stress treatment. This increase in adhesion rate may be attributed to the altered osmotic pressure inside and outside the protoplasts caused by saline stress, which aids in maintaining cellular integrity. However, the saline - alkali stress solution may also affect various organelles within the protoplasts, leading to enhanced cell adhesion.

### Effect of incubation at varying temperatures on the stability of endogenous beta-actin protein in protoplasts

**Fig. 4:**
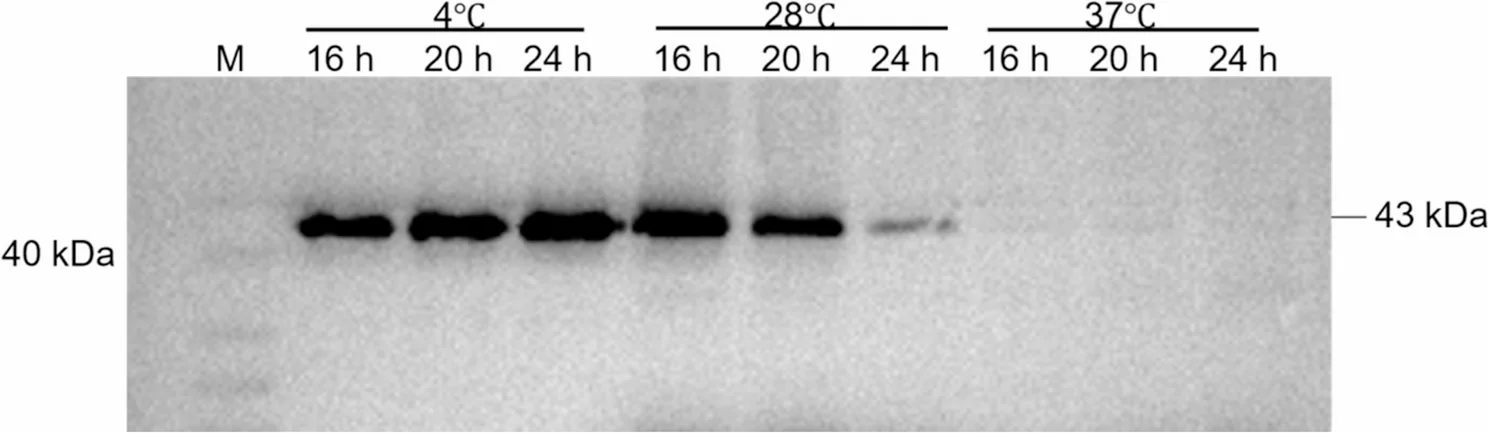
The expression of actin in maize protoplasts was analyzed by Western blot under different incubation temperatures and durations. M: marker. Each lane was loaded with 10 µL of protein sample

We further examined the expression of the endogenous protein β-actin in protoplasts following prolonged incubation. Protoplasts were incubated at 4 °C, 28 °C, and 37 °C, and the effect of temperature on these proteins was assessed using β-actin as a marker. The results indicate that β-actin expression remained stable at 4 °C for 16 to 24 h, with no significant decrease observed over the extended incubation period. In contrast, at 28 °C, β-actin expression gradually declined with increasing incubation duration, with a significant reduction in protein content observed at 24 h. At 37 °C, β-actin was nearly undetectable after 16 to 24 h of incubation. We hypothesize that these observations may be attributed to the impact of temperature on protoplast cell activity, leading to destabilization of endogenous proteins and their subsequent degradation (Fig. 4).

## Discussion

Foliar protoplasts isolated from maize leaves are a valuable resource for real-time detection of gene expression patterns and subcellular protein localization. They also provide a robust tool for investigating gene expression and function under specific environmental conditions [29]. In this study, we used a simple method to isolate protoplasts from maize leaves and examined the effects of different incubation durations on protoplast integrity and activity under high-temperature, low-temperature, and saline-alkaline stress conditions. Our findings establish an experimental foundation for understanding plant cell survival strategies in extreme temperatures. They also have significant theoretical and practical implications for optimizing protoplast preparation and culture conditions. Moreover, these results may enhance the potential applications of protoplasts in plant biotechnology under stress conditions. By analyzing β-actin protein expression in protoplasts under various stress conditions, we laid the groundwork for further comprehensive investigations into the molecular mechanisms of plant adaptation to temperature stress. Optimizing the temperature conditions during isolation can increase yields and improve the viability of isolated protoplasts, which is crucial for downstream applications such as genetic transformation and transient expression systems in plant biotechnology [30]. In this study, we found that maize protoplasts are sensitive to temperature. During the initial incubation period of 0 to 16 h, protoplasts appear to be in an acclimatized state [31]. As incubation time extends, the impact of temperature on protoplasts becomes more pronounced. At 4 °C, protoplasts exhibit relative stability over prolonged incubation (Figs. 1c and 2b, and 3). Incubation at 28 °C for 16 h has minimal effects on protoplasts; however, extended incubation leads to a significant decrease in cell number and an increase in cell adhesion rate (Figs. 1b and 2a, and 3). In contrast, incubation at 37 °C for 16 h severely impacts protoplasts, with intact cells becoming rare and aggregating into clusters under microscopic examination (Figs. 1d and 2c, and 3). Oxidative stress indicators were higher at 4 °C compared to 28 °C and 37 °C across various incubation times. Low temperatures may induce oxidative damage in maize protoplasts by overproducing reactive oxygen species (ROS), disrupting antioxidant system function, and inhibiting repair mechanisms. In contrast, cells at elevated temperatures (28 °C and 37 °C) may maintain redox homeostasis through more efficient metabolic and antioxidant responses. This highlights the oxidative stress induced by low temperatures and its complex effects on cellular metabolism. Liu et al. elucidated the salt tolerance mechanisms in Gossypium arboreum by conducting transcriptome profiling of root tips and root tip-derived protoplasts. Their research revealed that certain mechanisms, when disrupted, can lead to increased lipid peroxidation and impaired antioxidant activity under salt stress conditions [32]. Since protoplasts are highly sensitive to external stimuli due to the absence of the protective cell wall, selecting appropriate incubation temperatures and durations based on specific experimental goals is essential when using protoplasts as a model system. Our results indicate that a low-temperature environment has minimal adverse effects on maize protoplasts and may even provide a favorable survival condition for these cells.

In addition, the variation in protoplast yields at 0 h most likely arises from inconsistencies in manual leaf cutting, enzymatic digestion efficiency, and hemocytometer-based counting, rather than from true biological differences. To reduce these variations, we propose standardizing technical procedures by maintaining consistent methods for leaf cutting and enzymatic digestion, as well as utilizing automated cell counting devices to enhance accuracy and minimize human error. This finding could offer new insights for future research on low-temperature stress. At 4 °C protoplasts remained metabolically quiescent without measurable stress; this temperature therefore serves as a non-damaging baseline rather than a chilling stress, and provides an extended window for downstream manipulations. Temperature optimization is crucial for maize protoplast isolation. Compared to incubation at 28 °C and 37 °C, incubation at 4 °C better preserves plasma membrane integrity, minimizes cell adhesion, and results in the smallest increase in MDA levels (Fig. 3b). This finding is consistent with previous reports indicating that 4 °C effectively reduces lipid peroxidation and maintains membrane stability during short-term cold storage of plant and animal cells [33]. In contrast, 28 °C supports longer functional assays but significantly reduces viability beyond 16 h. At 37 °C, protoplasts rapidly aggregate and lyse, rendering this temperature unsuitable for any incubation protocol. For applications such as transformation or omics analyses, a “goal-oriented” selection of temperature and incubation duration is essential. This should be combined with standardized leaf-cutting, enzymatic digestion, and automated counting procedures to minimize initial variation and ensure experimental reproducibility.

Taken together, our data resolve the apparent contradiction between “stability” and “oxidative burst” at 4 °C by separating structural integrity from metabolic signalling. Short-term (≤ 16 h) storage at 4 °C is preferable for maize mesophyll protoplasts: viability remains ≥ 90%, membrane leakage < 5% and MDA does not rise, corroborating Zhang [34]. who stored maize protoplasts at 4 °C for 24 h without loss of electroporation competence. The transient 1.4-fold increase in H_2_O_2_ observed here is a mild, reversible priming response mediated by plasma-membrane NADPH oxidase, analogous to the rapid ROS burst that triggers CBF-dependent cold acclimation in Arabidopsis [35]. Thus, 4 °C is best interpreted as a maintenance condition rather than an injury-inducing chilling treatment during the first 16 h. For longer experiments (> 16 h) requiring transcriptional or enzymatic activity, 25–28 °C is more suitable because ROS return to baseline within 8 h and metabolic recovery is fastest. Thus, 4 °C provides a non-damaging maintenance window, whereas 25–28 °C supports full physiological function; the early ROS elevation is an adaptive signal rather than injurious oxidative stress.

The housekeeping gene actin is known for its stable transcript levels under various external stimuli, making it a commonly used internal reference gene in real-time fluorescence quantitative PCR experiments [36]. In our study, we observed that the levels of endogenous actin protein in protoplasts varied significantly under different temperature conditions (Fig. 4). Protoplasts are highly sensitive to temperature fluctuations, with elevated temperatures notably impairing cellular viability and increasing cell mortality. These effects may be attributed to changes in cellular integrity and functional activity in response to temperature variations, ultimately leading to the destabilization and degradation of endogenous proteins. Conversely, a low-temperature environment reduces the metabolic rate of protoplasts, decreases energy consumption, and extends cell survival. These conditions are more favorable for cell culture, enhancing cell viability and reducing non-specific cell adhesion. Research has shown that under low-temperature stress, plant cells can mitigate damage by modulating the levels of heat shock proteins (HSPs) and other molecular chaperones, which are crucial for maintaining proper protein folding and preventing aggregation [37]. Additionally, studies have elucidated a molecular mechanism where low-temperature signaling transcription factors (CBFs) interact with the light signaling transcription factor PIF3 to stabilize the red light and temperature receptor phyB, thereby enhancing plant freezing tolerance [36]. Our findings indicate that the integrity and activity of protoplast cells were effectively maintained during incubation at 4°C, with minimal impact observed as incubation time increased. The endogenous actin protein remained stable, suggesting that low temperatures had minimal effects on both protoplast cells and endogenous proteins (Fig. 4). Thus, during the first 16 h, 4°C should be regarded as a maintenance condition rather than an injurious chilling treatment. Importantly, because β-actin became highly unstable at 37°C, it is not a reliable loading control under severe stress. Similar stress-associated reductions in actin abundance have been reported under heat and oxidative stress in other systems. Therefore, equal loading was independently verified in this study, and β-actin was treated as a stress-responsive endogenous protein, not a constitutively stable reference. Temperature governs both the immediate viability and the oxidative trajectory of maize mesophyll protoplasts. Compared to 28°C and 37°C, incubation at 4°C preserved the highest number of intact cells, exhibited the lowest malondialdehyde (MDA) increase, and maintained the most stable actin band intensity (Fig. 4), confirming that membrane integrity and cytoskeletal proteins are best conserved under mild cold conditions. This finding aligns with previous reports indicating that 4°C slows phospholipid degradation and reduces NADPH-oxidase-mediated ROS bursts in maize and rice protoplasts, thereby extending the useful time window for transient expression [38, 39]. Conversely, incubation at 37°C caused rapid clumping and a threefold increase in hydrogen peroxide (H_2_O_2_) within 6 h, consistent with the heat-induced fluidization of the plasma membrane documented for Zea mays L. cv. B73 protoplasts at temperatures ≥ 35°C [40]. The intermediate temperature (28°C) provided a short optimum period (≤ 16 h), after which viability declined and cell adhesion increased; this mirrors the 15–18 h threshold observed in dicot systems [41], emphasizing that the routine °C” protocol cannot be extrapolated indefinitely. Our saline–alkali treatment (pH 9.1, 200 mM Na+) exacerbated temperature-dependent damage, but the 4°C condition still exhibited the slowest ROS accumulation and the smallest loss of SOD activity. Comparable Na_2_CO_3_ stress experiments in cotton protoplasts reported a 40% decrease in SOD activity after only 4 h at 28°C [36], whereas we recorded a 22% decrease at 4°C versus 65% at 28°C, underscoring that low temperature partially mitigates alkali-induced antioxidant collapse. Taken together, the data indicate that (i) 4°C provides a 16-h maintenance window, (ii) 28°C is suitable for short-term (< 12 h) functional assays, and (iii) 37°C should be avoided in maize protoplast workflows. The kinetic oxidative profile generated here offers a quantitative reference for future gene-editing or stress-signaling studies that use maize protoplasts as a single-cell model.

Our study investigated the effects of temperature and salt-alkali stress on maize protoplasts, focusing on protoplast production, viability, and protein expression. Similar studies have explored the impact of stress on protoplasts. For example, Ledoux et al. examined the effects of temperature stress on Arabidopsis thaliana protoplasts and found that temperature stress significantly affected protoplast viability [37]. Our results align with these findings, confirming that temperature stress has a substantial impact on protoplasts. However, our study uniquely combines temperature stress with salt-alkali stress, providing new insights into the combined effects of multiple stress factors on protoplasts.

## Conclusions

Our study investigated the effects of various temperature stresses and salt-alkali stress on maize protoplasts. The results demonstrated that both temperature and salt-alkali stress significantly impacted protoplast production, viability, and the expression of endogenous proteins. The analysis further indicates that 4 °C is suitable for short-term maintenance, 25–28 °C is appropriate for short-term functional assays, whereas 37 °C should be avoided because it rapidly compromises both protoplast integrity and endogenous protein stability. These findings provide valuable insights into the stress responses of protoplasts and establish a foundation for future research aimed at improving plant stress tolerance through protoplast-based techniques.

## Methods

### Extraction of protoplasts

Maize seedlings exhibiting yellowing, cultured in a dark environment, were harvested from the middle section of the second leaf of the maize inbred line B73 and used as experimental material. The leaves were cut into strips approximately 1.0 mm wide and transferred to Petri dishes containing an enzymatic solution (Fig. 1a).

The enzymatic solution contained 1.5% (w/v) cellulase R-10, 0.5% (w/v) macerozyme R-10, 0.4 M mannitol, 20 mM KCl, 20 mM MES, 10 mM CaCl_2_, and 1 mg mL^− 1^ BSA. The leaf strips underwent vacuum infiltration at a pressure of 30 kPa for 30 min, followed by enzymatic digestion for 4 h at room temperature on a shaker set to 40 rpm. An enzymatic termination solution (W5) was added in a volume equivalent to that of the enzymatic solution. The W5 solution contained sodium chloride (NaCl) (154 mM), calcium chloride (CaCl_2_) (125 mM), potassium chloride (KCl) (5 mM), and MES (2 mM). The resulting mixture was filtered through a 100-µm cell strainer and then centrifuged at 100×g for 2 min at 4 °C to pellet the protoplasts. The supernatant was removed, and 5 mL of W5 solution was added. The protoplast-containing solution was incubated on ice for 30 min, after which the protoplasts were pelleted again by centrifugation at 100×g for 2 min at 4 °C. The supernatant was discarded, and 1 mL of MMG solution (400 mM mannitol, 15 mM MgCl_2_, and 4 mM MES) was added. Finally, the protoplasts were resuspended in 500 µL of WI solution (20 mM KCl, 500 mM mannitol, and 4 mM MES) for subsequent experiments (Fig. 1a). The isolation procedure was performed with minor modifications from previously reported maize mesophyll protoplast protocols [41, 42].

### Temperature and saline-alkali treatment of protoplasts

For temperature treatments, maize protoplasts were resuspended in 1 mL of ice-cold W5 solution (154 mM NaCl, 125 mM CaCl_2_, 5 mM KCl, 2 mM MES, pH 5.8) and incubated in the dark at 4 °C, 28 °C and 37 °C for 0, 16, 20 and 24 h. In the saline stress experiment, a saline mixture of 200 mM (170 mM NaCl + 30 mM Na_2_CO_3_) was combined with the protoplasts in 1 mL of MMG solution. Subsequently, 500 µL of WI solution was added, and the mixture was incubated in the dark at 28 °C and 4 °C for 0, 16, 20, and 24 h, respectively.

### Microscopic observation and cell counting

Nuclear integrity (chromatin condensation) was evaluated using DAPI (28.50 µM, 3 min, in the dark at room temperature), while cytoplasmic viability was assessed separately with fluorescein diacetate (FDA). Subsequently, 10 µl of the protoplast suspension was aspirated and added to a hemocytometer for cell counting. Protoplast concentration was calculated using a standard hemocytometer formula. The cellular protoplast adhesion rate = the number of protoplast pairs (2 or more) where adhesion occurred/the total number of protoplasts observed.

### Extraction of total proteins from protoplasts and western blot analysis

To prepare lysed protoplast samples, add 200 µL of cell lysate (Western blotting and immunoprecipitation lysate, Beyotime P0043-100 mL) to 250 µL of protoplasts. The mixture should be shaken thoroughly and incubated on ice for 10 min. Subsequently, centrifuge the mixture at 12,000 rpm for 10 min at 4 °C. The total protein concentration of the extracted proteins was determined using the BCA Protein Quantification Kit.

For Western blot analysis, no immunoprecipitation was performed; whole-cell lysates were used directly for SDS-PAGE. Protein concentration was normalized by BCA assay, and 20 µg total protein was loaded per lane. The protein samples were initially separated using sodium dodecyl sulfate polyacrylamide gel electrophoresis (SDS-PAGE) and subsequently transferred to a polyvinylidene difluoride (PVDF) membrane under an electric current. The membrane is subsequently incubated with a primary antibody specific to the target protein (ABclonal stock number: AS029 HRP Rabbit Anti-Mouse IgG H + L). After washing with 1× TBST, the membrane is incubated with a secondary antibody (ABclonal No. AS003, HRP-conjugated goat anti-mouse IgG light chain). The β-actin bands were visualized using a color development solution (Thermo Fisher catalog number LC2671) and imaged with a gel imaging system (Tanon-2500).

### Detection of Superoxide Dismutase (SOD), Malondialdehyde (MDA), Reactive Oxygen Species (ROS), and Hydrogen Peroxide (H_2_O_2_) in protoplasts

After 250 µL of protoplasts were subjected to temperature stress and prolonged incubation, they were lysed using a plant Western blotting immunoprecipitation (IP) lysate to obtain a crude enzyme solution. This method was chosen because protoplasts lack a cell wall, making them more susceptible to lysis with specialized reagents. The selected lysate effectively disrupts the protoplast membranes, releasing intracellular components, including proteins and enzymes.

The activity of superoxide dismutase (SOD) in the crude enzyme solution was assessed using an SOD assay kit (BOXBIO No. AKAO001M-100 S), with absorbance measured at 560 nm using a spectrophotometer. The hydrogen peroxide (H_2_O_2_) content in the crude enzyme solution was analyzed using an H_2_O_2_ assay kit (Griseo Bio Art. No. G0168W48), with detection performed at a wavelength of 510 nm through zymography.

Percent inhibition = (ΔA blank − ΔA assay)/ΔA blank × 100%.

The inhibition percentage should be maintained between 30% and 70%. If the inhibition exceeds 70%, the crude enzyme solution should be re-diluted. If the inhibition is below 30%, the amount of crude enzyme solution used should be increased.

SOD (U/10^4^ Cell) = (Percent inhibition × Total Inverse × Total V Sample × D)/ (1 − Percent inhibition) × 500 × V Sample = 0.02 × Percent inhibition × D/ (1 − Percent inhibition).

This context, the following variables are defined: V_{\text{sample}} represents the volume of crude enzyme solution added to the reaction system (0.02 mL); V_{\text{reverse total}} denotes the total volume of the reaction system (0.2 mL); V_{\text{sample total}} indicates the total volume of the crude enzyme solution (1 mL); 500 refers to the number of cells (in tens of thousands); and D signifies the dilution factor of the crude enzyme solution, where D = 1 indicates that the crude enzyme solution is undiluted.

H_2_O_2_ content (nmol/10^4^ cells) = [(ΔA + 0.0115)/14.429]/ (500 × V1/V) × 10^3^ × D = 1.39 × (ΔA + 0.015) × D.

Definitions and Calculations The equation is defined as \Delta A = \Delta \text {Assay} - \Delta \text {Blank}, where the condition 0.01 < \Delta A < 1 must be satisfied. In this context: • V_1 represents the volume of the sample introduced into the reaction system (0.1 mL). • V denotes the volume of the extract added (1 mL).• 500 indicates the number of cells (expressed in tens of thousands). • D refers to the dilution factor of the crude enzyme solution, which is applicable only if the crude enzyme solution has not been diluted.

Reactive Oxygen Species (ROS) Detection Reactive oxygen species (ROS) detection was conducted on protoplasts subjected to high- and low-temperature stresses using the Reactive Oxygen Species Kit (BOXBIO Item No. AKCE002-1). A DCFH-DA fluorescent probe was employed to quantify intracellular ROS levels by measuring changes in fluorescence intensity. Fluorescence microscopy was used to visualize the fluorescence signal. The fluorescence intensity was measured at each time point before and after stimulation using a spectrophotometer with an excitation wavelength of 488 nm and an emission wavelength of 525 nm.

Malondialdehyde (MDA) Analysis Extracted protoplasts were lysed using Western blotting and immunoprecipitation (IP) cell lysates to obtain a crude enzyme solution following temperature stress and prolonged incubation. The crude enzyme solution was analyzed using a Malondialdehyde (MDA) Kit (BOXBIO No. AKFAO13C), and the absorbance values were measured at 450 nm, 532 nm, and 600 nm using a spectrophotometer.

MDA content (nmol/10^4^ cells) = (6.45 × (ΔA532 − ΔA600) − 1.29 × ΔA450) × Total V/ (500 × V sample/V mentioned).

Here, V_{\text{total}} represents the total volume of the system (1 mL), V_{\text{sample}} denotes the volume of the sample to be tested in the reaction system (0.2 mL), V_{\text{lift}} indicates the volume of the extract (1 mL), and 500 refers to the.

Where V total represents the total volume of the system (1 mL), V sample denotes the volume of the sample to be tested in the reaction system (0.2 mL), V lift indicates the volume of the extract (1 mL), and 500 refers to the number of cells in ten thousand.

## Supplementary Information

Supplementary Material 1.

## Data Availability

Not applicable.

## Declarations

The statements, opinions and data contained in all publications are solely those of the individual author(s) and contributor(s) or the editor(s) disclaim responsibility for any injury to people or property resulting from any ideas, methods, instructions or products referred to in the content.
